# Phenotypic evaluation of local rice (*Oryza sativa* L.) landraces for tolerance to submergence stress

**DOI:** 10.1371/journal.pone.0357048

**Published:** 2026-08-25

**Authors:** Samir Regmi, Binita Poudel, Bikalpa Neupane, Pawan Chapagaee, Sagar Lamsal

**Affiliations:** 1 Gokuleshwor Agriculture and Animal Science College, IAAS, Tribhuvan University, Baitadi, Nepal; 2 Department of Bioscience and Biotechnology, Fukui Prefectural University, Fukui, Japan; KGUT: Graduate University of Advanced Technology, IRAN, ISLAMIC REPUBLIC OF

## Abstract

Rice seedlings are often subjected to abiotic stresses, particularly flooding and submergence in lowland areas, highlighting the need to develop genotypes with improved tolerance to these conditions. This study aimed to evaluate the submergence tolerance of seven rice genotypes and identify adaptive traits associated with survival under complete submergence. Seven genotypes were phenotypically evaluated under complete submergence (155 cm water depth, 10 days) in a Completely Randomized Design (CRD) with nine replications. After a seven-day recovery period, the landraces were evaluated for survival, shoot elongation, biomass accumulation, green leaf retention, and chlorophyll content. All variables across stages showed significant genotypic variation (P ≤ 0.001), suggesting that the landraces used different adaptive methods. Sawa Mansuli Sub-1 and Chamse 6-mahine showed minimal elongation, superior leaf retention, higher chlorophyll content, and significantly greater survival; excessive shoot elongation, especially in Chamse 3-mahine, was linked to high biomass accumulation during submergence but highest mortality after recovery. Principal component analysis distinguished quiescence-type landraces (Sawa Mansuli Sub-1, Chamse 6-mahine, and Seto Bikashi) from susceptible escape-type landraces, with PC1 and PC2 explaining 95.8% of total variation. Correlation analysis revealed a negative association between shoot elongation and survival and positive associations between survival and post-recovery biomass, green leaf retention, and chlorophyll content. Although field testing and genetic research are still required, these results indicate that Chamse 6-mahine and Seto Bikashi are promising local rice varieties for creating flood-tolerant rice. Furthermore, they highlight quiescence-based energy conservation, chlorophyll content, and leaf viability as important determinants of submergence tolerance.

## 1. Introduction

Rice (*Oryza sativa* L*.*) is a staple food for about 90 percent of the Asian population and contributes substantially to global food and nutritional security [1]. In Nepal, rice is a vital part of the country’s diet; it is an important contributor to agricultural GDP and provides employment [2]. Sustaining and increasing rice productivity under changing climatic conditions is therefore critical for ensuring food security in Asia’s rice-based economies. Among the various abiotic stresses affecting rice cultivation, flooding and submergence have emerged as serious threats to rice production, particularly in lowlands and rainfed ecosystems [3].

The submergence conditions affect the normal growth and development of the plant, especially at an early age. Complete or prolonged submergence restricts the gas exchange, reduces light interception, impairs photosynthesis, and accelerates carbohydrate depletion, often resulting in plant mortality and severe yield loss [4]. In severe events, yield losses can reach 80–90%, whereas submergence-tolerant varieties carrying the *SUB1* locus can survive for approximately 7–14 days of complete flooding (Khanal et al., 2024). The submergence conditions due to flooding reduce the world rice production by 4.3% [5], and in Nepal it is even more, an 8.74% yield reduction due to flooding and submergence [6]. Unlike partial waterlogging, complete submergence severely restricts oxygen diffusion and photosynthesis, making it one of the most destructive flooding stresses affecting rice [7]. However, more intense and erratic rainfall patterns associated with climate change are expected to increase the frequency and severity of submergence events in many rice‑growing regions, particularly where heavy rainfall coincides with inadequate drainage [8], highlighting the need for varieties with improved tolerance.

Rice shows substantial genotypic variation in response to flooding, reflecting underlying differences in physiological and genetic adaptation [9]. Two major adaptive strategies have been identified in response to complete submergence: the escape and quiescence strategies. In the escape strategy, plants rapidly elongate shoots and internodes to restore contact with the atmosphere, thereby facilitating gas exchange above the water surface, but often at the cost of excessive energy consumption and lodging risk [10]. In contrast, the quiescence strategy involves suppressing elongation growth, allowing plants to conserve energy and carbohydrate reserves during submergence that support plant survival and post-submergence recovery [9]. The *SUB1* gene derived from the locus from the Indian landrace FR13A underpins the quiescence strategy, and *SNORKEL1* (SK1) and *SNORKEL2* (SK2) genes are responsible for the escape strategy [11]. These contrasting strategies involve differential regulation of hormonal pathways, including ethylene, gibberellins, and abscisic acid [12,13]. Knowledge of these adaptive mechanisms has facilitated the development of submergence-tolerant cultivars. However, the narrow genetic base of many modern varieties emphasizes the value of traditional landraces as potential sources of novel tolerance alleles [14]. However, empirical data on the survivability and phenotypic plasticity of locally adapted landraces under standardized submergence conditions remain limited, particularly in South Asian flood‑affected regions such as Nepal. This knowledge gap constrains the effective use of local germplasm in breeding programs aimed at developing climate‑resilient varieties tailored to specific agroecosystems.

Molecular and breeding studies have greatly advanced submergence tolerance by introgressing loci such as *SUB1* into high-yielding backgrounds. Nevertheless, evaluating locally adapted landraces remains essential for identifying additional phenotypic sources of tolerance [15]. Environmental variation often complicates field screening for submergence tolerance. Submergence experiments provide a reliable phenotyping platform by guaranteeing consistent stress application and maintaining the homogeneity [16]. These methods improve the accuracy of distinguishing genotypes [17]. While submergence tolerance has been extensively researched, there is very limited study conducted to evaluate the performance of local landraces under the complete submergence conditions, especially in flood-prone agroecosystems [18]. In this context, the present study aims to phenotypically evaluate local rice landraces for tolerance to complete submergence, followed by recovery, focusing on shoot elongation, biomass dynamics, green leaf retention, and post‑recovery chlorophyll content. By comparing these phenotypic responses, we aim to identify landraces that exhibit relatively greater submergence tolerance under controlled conditions and to provide candidate germplasm for future field evaluation and breeding of climate-resilient rice adapted to flood‑prone environments.

## 2. Materials and methods

### 2.1. Experimental site

The experiment was conducted in a laboratory of Gokuleshwor Agriculture & Animal Science College, Baitadi, Nepal, during July of 2025. All of the seeds used in this study were collected from local farmers in Gokuleshwor, Baitadi, Nepal, with their verbal consent. No protected or endangered plant species were used, and no field permit was required for the collection of landraces. The study was conducted in accordance with standard agronomic research protocols at Tribhuvan University, Nepal’s Institute of Agriculture and Animal Science (IAAS). This kind of controlled agronomic experiment did not require institutional ethical approval. The site is located between at 29°39’ 47” N latitude & 80°32’33” E longitude, at an, altitude of 810 masl. The submergence tolerance of rice landraces was evaluated by monitoring phenotypic changes in pot-grown plants that were subjected to complete inundation under controlled conditions.

### 2.2. Plant materials

Seven genotypes of rice were used for this study; out of these, one is submergence tolerant as a control, and the other six are local landraces collected from farmers (Table 1).

**Table 1:** List of rice genotypes used in the experiment.

Treatments	Genotypes	Type
T_0_ (Control)	Sawa Mansuli sub-1	Released
T_1_	Bhure Dhan	Landrace
T_2_	Rato Bikashi	Landrace
T_3_	Local Basmati	Landrace
T_4_	Seto Bikashi	Landrace
T_5_	Chamse 3-mahine	Landrace
T_6_	Chamse 6-mahine	Landrace

Seeds were collected from the farmers of Gokuleshwor, Baitadi, Nepal, ensuring representation of traditional genetic diversity.

### 2.3. Experimental design

#### Seed sterilization and germination.

Seeds of all genotypes were surface sterilized using 3% sodium hypochlorite (NaOCl) solution for five minutes to remove surface contaminants. After sterilization, seeds were thoroughly rinsed by using distilled water eight times [19]. Sterilized seeds were placed on moist blotting paper in petri plates and incubated at room temperature (22 ± 3°C) for 6 days to allow for germination. Uniformly germinated seeds were selected for the transplanting.

#### Pot preparation and planting.

Soil used for study was taken from an agricultural field, allowed to air dry, and then sieved to remove coarse particles, stones, and debris. To enhance nutrient availability and promote seedling establishment, well-decomposed FYM was thoroughly mixed with the soil at a rate equal to 10 t ha^−1^. Plastic pots were filled with the prepared growth medium (4 kg pot^−1^). Using sterile tweezers, pre-germinated seedlings were planted while maintaining a plant population of four plants per pot, with nine replications per treatment.

#### Pre-submergence growth conditions.

Transplanted seedlings were grown for 14 days in natural (day and night) conditions. Throughout this period a uniform water depth of 5 cm was maintained. At 14 DAT, the height of the whole population was measured, and the first three replications were destructively sampled to measure fresh and dry biomass (n = 3 per geontype).

#### Submergence treatment.

At 14 DAT, the remaining six replications were subjected to complete submergence in the tank by maintaining the water depth of 155 cm. A depth of 155 cm was used to ensure complete submergence of all genotypes throughout the 10-day submergence period. Instead of replicating a particular field flood depth, the experiment’s goal was to create a controlled complete submergence under uniform conditions [20]. Plants were subjected to submergence for 10 days, and throughout these 10 days, a constant water level was maintained to ensure complete plant submergence.

#### Post-submergence.

Upon completion of the submergence treatment, plants were returned to their original growth conditions and allowed a seven-day recovery period. A plant was considered viable when at least one new leaf emerged during recovery. Again, we destructively sampled the three replications to measure fresh biomass data. After this we placed the remaining three replications under normal conditions and allowed them to recover for 7 days. Throughout these 7 days we followed all the agronomic practices properly and prevented the plant from suffering from any other stress conditions.

#### After recovery.

After the 7 days under normal conditions, we recorded data on the number of green leaves per plant. Because apical meristems were damaged during submergence and new leaves appeared at varying rates, standardized height measurements were unreliable; thus, plant height was not measured after recovery. As a result, post-recovery height was excluded from multivariate analysis. The remaining three replications were destructively sampled to measure post-recovery biomass, and leaf tissue was collected for chlorophyll analysis (n = 3 per genotype).

Nine biological replicates were established for each of the seven rice genotypes at the beginning of the experiment. At 14 days after transplanting (DAT), prior to submergence, plant height was measured in all nine replicates, after which three randomly selected replicates were destructively harvested for biomass determination. The remaining six replicates were subjected to complete submergence for 10 days. Following de-submergence, all six replicates were assessed for the designated non-destructive parameters, and three of these replicates were subsequently destructively sampled for biomass determination. The remaining three replicates were maintained under normal growing conditions for a 7-day recovery period, after which all recovery-related traits, including survival, green leaf number, chlorophyll content, fresh weight, and dry weight, were recorded. Accordingly, statistical analyses for recovery-phase variables were performed using the three biological replicates remaining after the sequential destructive sampling.

#### Measured parameters.

Plant height was measured before submergence (14 DAT) and immediately after the 10-day submergence period, starting from the soil’s surface and ending at the tip of the tallest leaf. Height after submergence (HAS) was used to calculate shoot elongation rate and was included in PCA and correlation analysis. Weighing balances were used to measure biomass at three different stages: prior to submergence, following submergence, and following regrowth. After drying plant specimens in a hot air oven at 65°C until they reached a consistent weight, dry biomass was measured using a weighing machine. Three phases of data collection were made: prior to submergence, following submergence, and following regrowth. At each measurement stage, all fully green (non-senesced) leaves were counted to calculate the number of green leaves per plant. If a leaf’s entire lamina still had a noticeable green color, it was considered fully green. Due to the difficulty of differentiating senescence stages in fully submerged settings, partial viability was not evaluated. At the completion of the seven-day recovery period, the leaf of all plants was used to assess the chlorophyll concentration using the spectrophotometric procedure given by [21].

#### Shoot elongation rate (cm/day).

Calculated to assess the escape response of plants during submergence [22].


$$\begin{array}{c} Shoot\;Elongation\;Rate\;(\frac{cm}{day})=\frac{Height\;after\;submergence-Height\;before\;submergence}{\text{Duration of Submergence }(\text{days})} \end{array}$$

#### Survival percentage.

Calculated after the recovery phase to know landrace character [22].


$$\begin{array}{c} Survival\;\%=\frac{\text{Number of surviving plants after recovery }(\text{per pot})}{\text{Number of plants before submergence }(\text{per pot})}\times \text{1}\text{0}\text{0} \end{array}$$

#### Statistical analysis.

A completely randomized design (CRD) and one-way analysis of variance (ANOVA) were used to assess the impact of submergence stress on various rice landraces. To make sure the ANOVA assumptions were met, the data were examined for homogeneity of variance and normality before analysis. Tukey’s Honest Significant Difference (HSD) test was used to compare treatment means at the 5% significance level. All measured variables were evaluated with principal component analysis (PCA) using a correlation matrix in order to examine multivariate relationships among morpho-physiological traits. Pairwise relationships between traits were evaluated using Pearson’s correlation coefficients. RStudio was used for all statistical analyses. At P ≤ 0.05, differences were considered statistically significant. Post-recovery assessments were limited to n = 3 replications per genotype because of the destructive sampling methodology. Despite the fact that the recovery-phase sample size (n = 3) limits statistical power, this design was implemented uniformly across all genotypes and was the result of distructive sampling necessary for biomass and chlorophyll measurements. All post-recovery traits showed extremely significant variations (P ≤ 0.001) and resulitng ANOVA degrees of freedom (F_(6,14)_) are statistically valid.

## 3. Results

### 3.1. Before Submergence

#### 3.1.1. Plant height.

Analysis of variance (ANOVA) showed highly significant variation (F _(6,56)_ = 81.38, P ≤ 0.001). The mean height of plants among the treatments has significant variation. The highest plant height was recorded in Chamse 3-mahine (46.02 ± 0.9 cm), followed by Local Basmati (37.1 ± 0.74 cm). Rato Bikashi (36.7 ± 0.77 cm), Bhure Dhan (36.7 ± 0.36 cm), and Seto Bikashi (35.4 ± 0.72 cm) show intermediate variation, which was statistically similar to each other. Low variation in plant height was shown by Chamse 6-mahine (30.1 ± 0.43 cm) and Sawa Mansuli Sub-1 (27.4 ± 0.44 cm) (Table 2).

**Table 2:** Initial growth characteristics of rice varieties before submergence stress.

Treatment	Plant height (cm)	Fresh weight (g)	Dry weight (g)
Sawa Mansuli Sub-1	27.4 ± 0.45^c^	0.19 ± 0.02^d^	0.02 ± 0.002^d^
Bhure Dhan	36.2 ± 0.36^b^	0.35 ± 0.03^bc^	0.04 ± 0.003^bc^
Rato Bikashi	36.7 ± 0.75^b^	0.48 ± 0.03^a^	0.05 ± 0.004^a^
Local Basmati	37.1 ± 0.75^b^	0.41 ± 0.02^ab^	0.04 ± 0.003^ab^
Seto Bikashi	35.4 ± 0.72^b^	0.45 ± 0.02^ab^	0.05 ± 0.001^ab^
Chamse 3-mahine	46.03 ± 0.9^a^	0.5 ± 0.03 ^a^	0.05 ± 0.001^ab^
Chamse 6-mahine	30.1 ± 0.44^c^	0.28 ± 0.03 ^cd^	0.03 ± 0.004 ^cd^
Grand Mean	35.56	0.38	0.04
CV	5.53	11.55	13.28
MSD	2.83	0.12	0.015
F-test	***	***	***

#### 3.1.2. Fresh weight.

Fresh biomass varied significantly among genotypes (F _(6,14)_ = 19.62, P ≤ 0.001). The highest fresh biomass accumulation after 14 days of plating was seen in Chamse 3-mahine (0.503 ± 0.003 g), followed by Rato bikashi (0.48 ± 0.03 g). Chamse 6-mahine (0.27 ± 0.035 g) showed lower fresh biomass accumulation, whereas Sawa Mansuli Sub-1 (0.197 ± 0.018 g) showed the lowest fresh biomass accumulation (Table 2).

#### 3.1.3. Dry weight.

Dry biomass also varied significantly across treatments (F _(6,14)_ = 16.93, P ≤ 0.001). Out of seven landraces Rato Bikashi (0.056 ± 0.004 g) has the highest dry mass accumulation, followed by Chamse 3-mahine (0.053 ± 0.001 g). Bhure Dhan (0.04 ± 0.003 g) has intermediate dry biomass accumulation, which is statistically similar to Seto Bikashi (0.048 ± 0.001 g) and Local Basmati (0.043 ± 0.003 g). Chamse 6-mahine (0.028 ± 0.004 g) and Sawa Mansuli Sub-1 (0.020 ± 0.002 g) have the lowest dry mass accumulation out of all the treatments (Table 2).

### 3.2. After submergence

#### 3.2.1. Plant height.

Significant genotypic differences were observed in plant height following ten days of submergence (F _(6,35)_ = 129.7, P ≤ 0.001). The highest plant height after the submergence was observed in Chamse 3-mahine (97.3 ± 1.75 cm), followed by Local Basmati (66.9 ± 1.77 cm). Intermediate variance was shown in Bhure Dhan (64.1 ± 1.72 cm), Rato Bikashi (58.4 ± 2.34 cm), and Seto Bikashi (56.4 ± 1.22 cm). Lower plant height was recorded in Chamse 6-mahine (50.2 ± 1.35 cm), and the lowest height was recorded in Sawa Mansuli Sub-1 (37.0 ± 0.92 cm) (Table 3).

**Table 3:** Effect of submergence stress on plant height, shoot elongation, leaf retention, and biomass of rice varieties during submergence.

Treatment	Plant height (cm)	Shoot elongation (cm/day)	Green Leaves	Fresh weight (g)	Dry weight (g)
Sawa Mansuli Sub-1	37.0 ± 0.92^f^	0.93 ± 0.05^e^	3.96 ± 0.18^a^	0.97 ± 0.03^c^	0.10 ± 0.01^c^
Bhure Dhan	64.1 ± 1.72^bc^	2.79 ± 0.18^bc^	1.46 ± 0.17^c^	1.96 ± 0.12^b^	0.20 ± 0.01^b^
Rato Bikashi	58.4 ± 2.34 ^cd^	2.22 ± 0.2 ^cd^	1.25 ± 0.17^c^	1.58 ± 0.09^bc^	0.16 ± 0.009^bc^
Local Basmati	67.0 ± 1.77^b^	3.08 ± 0.16^b^	1.38 ± 0.12^c^	2.05 ± 0.04^b^	0.21 ± 0.003^b^
Seto Bikashi	56.4 ± 1.22^de^	1.86 ± 0.12^d^	2.75 ± 0.17^b^	1.44 ± 0.12^bc^	0.14 ± 0.02^bc^
Chamse 3-mahine	97.3 ± 1.75^a^	6.22 ± 0.24^a^	1.17 ± 0.08^c^	4.17 ± 0.23^a^	0.42 ± 0.02 ^a^
Chamse 6-mahine	50.2 ± 1.35^e^	0.49 ± 0.13^e^	3.04 ± 0.12^b^	1.24 ± 0.1^c^	0.12 ± 0.009^c^
Grand Mean	61.47	2.51	2.14	1.91	0.19
CV	6.52	16.22	15.93	12.25	14.19
MSD	7.23	0.73	0.61	0.65	0.07
F-test	***	***	***	***	***

#### 3.2.2. Shoot elongation.

Shoot elongation rate during the 10-day submergence period differs markedly among the genotypes (F _(6,35)_ = 127.6, P ≤ 0.001). Higher shoot elongation was shown in Chamse 3-mahine (6.22 ± 0.23 cm/day), followed by Local Basmati (3.08 ± 0.16 cm/day). Bhure Dhan (2.79 ± 0.18 cm/day), Rato Bikashi (2.22 ± 0.21 cm/day), and Seto Bikashi (1.86 ± 0.12 cm/day) showed intermediate shoot elongation. Lower shoot elongation was seen in Sawa Mansuli Sub-1 (0.93 ± 0.05 cm/day) and lowest in Chamse 6-mahine (0.49 ± 0.13 cm/day) (Table 3).

#### 3.2.3. Green leaves.

The number of green leaves per plant varies significantly among genotypes (F _(6,35)_ = 62.41, P ≤ 0.001). Maximum number of green leaves was seen in Sawa Mansuli sub-1 (3.96 ± 0.18), followed by Chamse 6-mahine (3.04 ± 0.12) and Seto Bikashi (2.75 ± 0.09). Bhure Dhan (1.46 ± 0.18) shows a lower green leaf number, which is statistically similar to Local Basmati (1.38 ± 0.12), and Rato Bikashi (1.25 ± 0.17). The lowest green leaves were seen in Chamse 3-mahine (1.17 ± 0.08) (Table 3).

#### 3.2.4. Fresh weight.

Post-submergence fresh biomass differed significantly among genotypes (F _(6,14)_ = 61.91, P ≤ 0.001). Chamse 3-mahine (4.17 ± 0.22 g) reported the highest mean fresh biomass, followed by Local Basmati (2.05 ± 0.04 g) and Bhure Dhan (1.96 ± 0.12 g). Rato Bikashi (1.58 ± 0.098 g) and Seto Bikashi (1.44 ± 0.19 g) reported intermediate fresh biomass accumulation, which is statistically similar as well. Low fresh biomass accumulation was observed in Chamse 6-mahine (1.24 ± 0.10 g) and Sawa Mansuli sub-1 (0.96 ± 0.029 g) (Table 3).

#### 3.2.5. Dry weight.

Data from the analysis of different variance shows very high significant variation (F _(6,14)_ = 46.88, P ≤ 0.001) among the different treatments in dry mass accumulation after the ten days of submergence. Chamse 3-mahine (0.42 ± 0.02 g) and Local Basmati (0.21 ± 0.003 g) show the highest dry mass accumulation; intermediate dry mass accumulation was observed in Bhure Dhan (0.20 ± 0.017 g), Rato Bikashi (0.16 ± 0.009 g), and Seto Bikashi (0.14 ± 0.021 g). Chamse 6-mahine (0.12 ± 0.009 g) and Sawa Mansuli Sub-1 (0.10 ± 0.01 g) show the lowest dry mass accumulation among all the treatments (Table 3).

### 3.3. After recovery

#### 3.3.1. Green leaves.

Chamse 3-mahine produced the lowest green leaves (0.08 ± 0.08) during recovery, while Sawa Mansuli sub-1 recorded the highest green leaves count during recovery (5.67 ± 0.22; F _(6,14)_ = 38.18, P ≤ 0.001), followed by Chamse 6-mahine (4.42 ± 0.44), and Seto Bikashi (2.33 ± 0.82). A lower number of green leaves was seen in Rato Bikashi (0.17 ± 0.17), which is statistically similar to Local Basmati (0.33 ± 0.17) and Bhure Dhan (0.25 ± 0.14). (Table 4).

**Table 4:** Growth performance, survival, and chlorophyll content of rice varieties after recovery from submergence stress.

Treatment	Green leaves	Fresh wt (g)	Dry wt (g)	Survival (%)	Chlorophyll A (μg/ml)	Chlorophyll B (μg/ml)	Chlorophyll A + B (μg/ml)
Sawa Mansuli Sub-1	5.67 ± 0.22^a^	4.72 ± 0.38^a^	0.49 ± 0.043^a^	100 ± 0^a^	2.61 ± 0.03^a^	2.83 ± 0.19^a^	5.44 ± 0.20^a^
Bhure Dhan	0.25 ± 0.14^c^	0.82 ± 0.06^c^	0.07 ± 0.006^c^	16.7 ± 8.33^c^	0.31 ± 0.06^d^	0.22 ± 0.03^c^	0.53 ± 0.09^c^
Rato Bikashi	0.17 ± 0.17^c^	0.63 ± 0.04^c^	0.06 ± 0.003^c^	8.33 ± 8.33^c^	0.16 ± 0.10^d^	0.32 ± 0.02^c^	0.48 ± 0.10^c^
Local Basmati	0.33 ± 0.17^c^	0.72 ± 0.02^c^	0.06 ± 0.02^c^	16.7 ± 8.33^c^	0.24 ± 0.11^d^	0.17 ± 0.01^c^	0.41 ± 0.12^c^
Seto Bikashi	2.33 ± 0.82^b^	2.42 ± 0.23^b^	0.24 ± 0.02^b^	66.7 ± 8.33^ab^	1.21 ± 0.20^c^	1.24 ± 0.35^b^	2.45 ± 0.51^b^
Chamse 3-mahine	0.08 ± 008^c^	0.39 ± 0.02 ^c^	0.03 ± 0.001^c^	8.33 ± 8.33^c^	0.09 ± 0.06^d^	0.12 ± 0.03^c^	0.22 ± 0.09^c^
Chamse 6-mahine	4.42 ± 0.44^a^	2.92 ± 0.12^b^	0.30 ± 0.005^b^	58.3 ± 8.33^b^	1.74 ± 0.07^b^	1.88 ± 0.19^b^	3.62 ± 0.27^b^
Grand Mean	1.89	1.80	0.18	39.28	0.91	0.96	1.87
CV	34.58	16.82	18.24	34.01	20.74	31.30	22.61
MSD	1.82	0.84	0.092	37.25	0.52	0.84	1.18
F-test	***	***	***	***	***	***	***

#### 3.3.2. Chlorophyll-a.

Sawa Mansuli Sub-1 had the highest post-recovery chlorophyll-a content (2.61 ± 0.03 µg/ml), followed by Chamse 6-mahine (1.74 ± 0.07 µg/ml) and Seto Bikashi (1.21 ± 0.20 µg/ml), indicating a higher capacity for photosynthetic recovery in these genotypes (F_(6,14)_ = 80.6, P ≤ 0.001; Table 4). The lowest chlorophyll-a (0.09 ± 0.06 µg/ml) was found in Chamse 3-mahine, which is consistent with its total post-submergence mortality.

#### 3.3.3. Chlorophyll-b.

Sawa Mansuli Sub-1 (2.83 ± 0.19 µg/ml) and Chamse 6-mahine (1.88 ± 0.19 µg/ml) recorded the highest values among the seven genotypes for chlorophyll-b content, which followed a pattern similar to that of chlorophyll-a (F_(6,14)_ = 36.23, P ≤ 0.001; Table 4).

#### 3.3.4. Total chlorophyll.

Though Sawa Mansuli Sub-1 (5.44 ± 0.20 µg/ml) accumulating the highest total and Chamse 3-mahine (0.22 ± 0.09 µg/ml) accumulating the lowest, total chlorophyll (a + b) correlated with the individual pigment trends, highlighting the strong correlation between post-recovery chlorophyll content and submergence tolerance (F_(6,14)_ = 68.28, P ≤ 0.001; Table 4).

#### 3.3.5. Fresh weight.

Anova revealed a highly significant variation (F _(6,14)_ = 84.9, P ≤ 0.001) among the treatments after the 10 days of submergence and 7 days of regrowth. The highest fresh weight was observed in Sawa Mansuli Sub-1 (4.72 ± 0.37 g), followed by Chamse 6-mahine (2.92 ± 0.01 g). The lowest fresh biomass was observed in Rato Bikashi (0.64 ± 0.04 g), which is statistically similar to Chamse 3-mahine (0.4 ± 0.02 g) (Table 4).

#### 3.3.6. Dry weight.

Submergence of rice plants for ten days and regrowth for seven days shows highly significant variation among the treatments (F _(6,14)_ = 80.16, P ≤ 0.001). Among the seven treatments, the highest dry biomass accumulation was seen in Sawa Mansuli Sub-1 (0.49 ± 0.04 g), followed Chamse 6-mahine (0.30 ± 0.005 g) and Seto Bikashi (0.25 ± 0.02 g). Bhure Dhan (0.07 ± 0.006 g) shows intermediate dry biomass accumulation, which is statistically similar to Local Basmati (0.064 ± 0.002 g) and Rato Bikashi (0.057 ± 0.003 g). The lowest dry mass accumulation was seen in Chamse 3-mahine (0.035 ± 0.001 g) (Table 4).

#### 3.3.7. Survival percentage.

Survival percentage after ten days of submergence and seven days of recovery differed significantly among genotypes (F _(6,14)_ = 27.93, P ≤ 0.001). The highest percentage of survival was seen in Sawa Mansuli Sub-1 (100 ± 0%), followed by Seto Bikashi (66.67 ± 8.33%) and Chamse 6-mahine (58.3 ± 8.33%). Lower survival was shown by Rato Bikashi (8.33 ± 8.33%) and Chamse 3-mahine (8.33 ± 8.33%) (Table 4).

### 3.4. Principal component analysis

**Fig 1:**
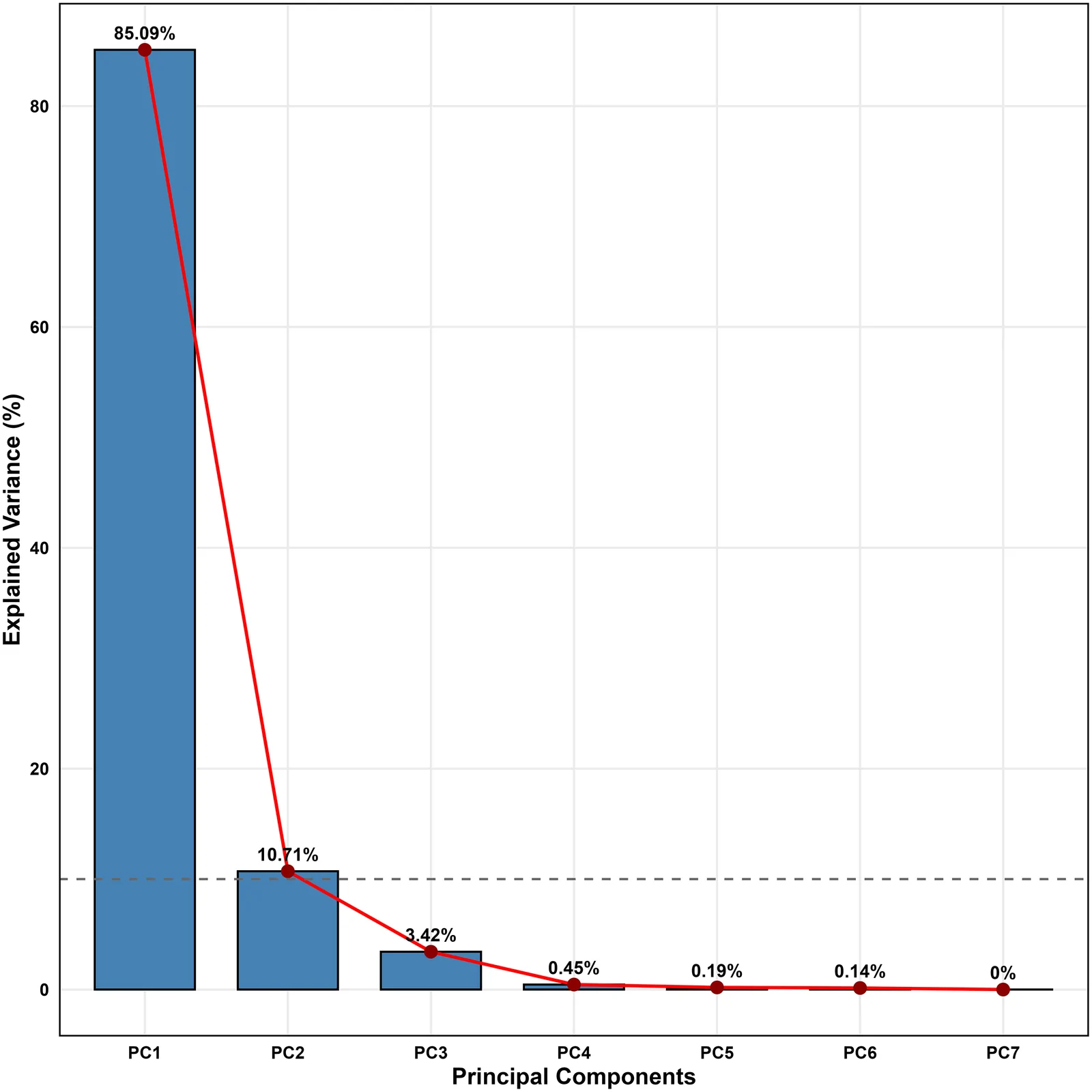
Scree plot showing the proportion of variance explained by principal components in rice landraces.

**Fig 2:**
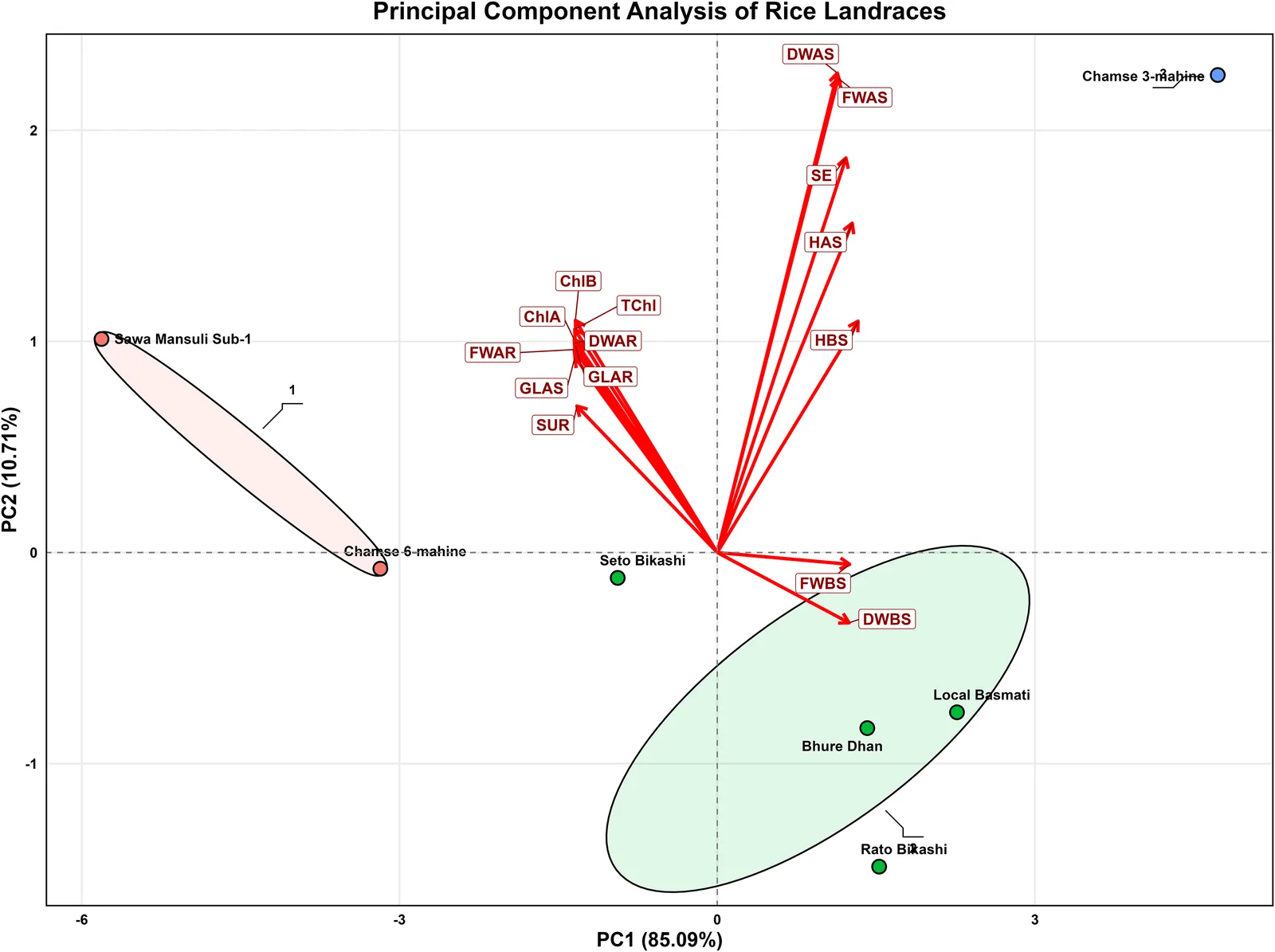
Principal Component Analysis (PCA) biplot showing the multivariate relationships among rice landraces and agro-morphological traits. HBS: Height Before Submergence, HAS: Height After Submergence, FWBS: Fresh Weight Before Submergence, FWAS: Fresh Weight After Submergence, DWBS: Dry Weight Before Submergence, DWAS: Dry Weight After Submergence, SE: Shoot Elongation, GLAS: Green Leaves After Submergence, GLAR: Green Leaves After Recovery, FWAR: Fresh Weight After Recovery, DWAR: Dry Weight After Recovery, SUR: Survival Percentage, ChlA: Chlorophyll-a, ChlB: Chlorophyll-b, TChl: Total Chlorophyll.

The multivariate relationship between morpho-physiological and biochemical characteristics linked to submergence tolerance in rice landraces was assessed using principal component analysis (PCA). Together, the first two principal components (PC1 and PC2) explained 95.80% of the total variation, with PC1 contributing 85.09% and PC2 contributing 10.71% (Fig 1). This suggests that the variability among the genotypes under study was sufficiently summarized by these components. The rice landraces were clearly separated according to how they responded to submergence stress, according to the PCA biplot. Sawa Mansuli Sub-1 and Chamse 6-mahine were located on the negative side of PC1, while genotypes like Local Basmati, Bhure Dhan, Rato Bikashi, and Seto Bikashi were grouped on the positive side, indicating different physiological and biochemical reactions under submerged conditions. Chamse 3-mahine had a distinct position in the upper positive quadrant, due to its great shoot elongation and high biomass accumulation during submergence, which are characteristics linked to an escape strategy and ultimately full post-submergence mortality (Fig 2).

### 3.5. Correlation analysis

**Fig 3:**
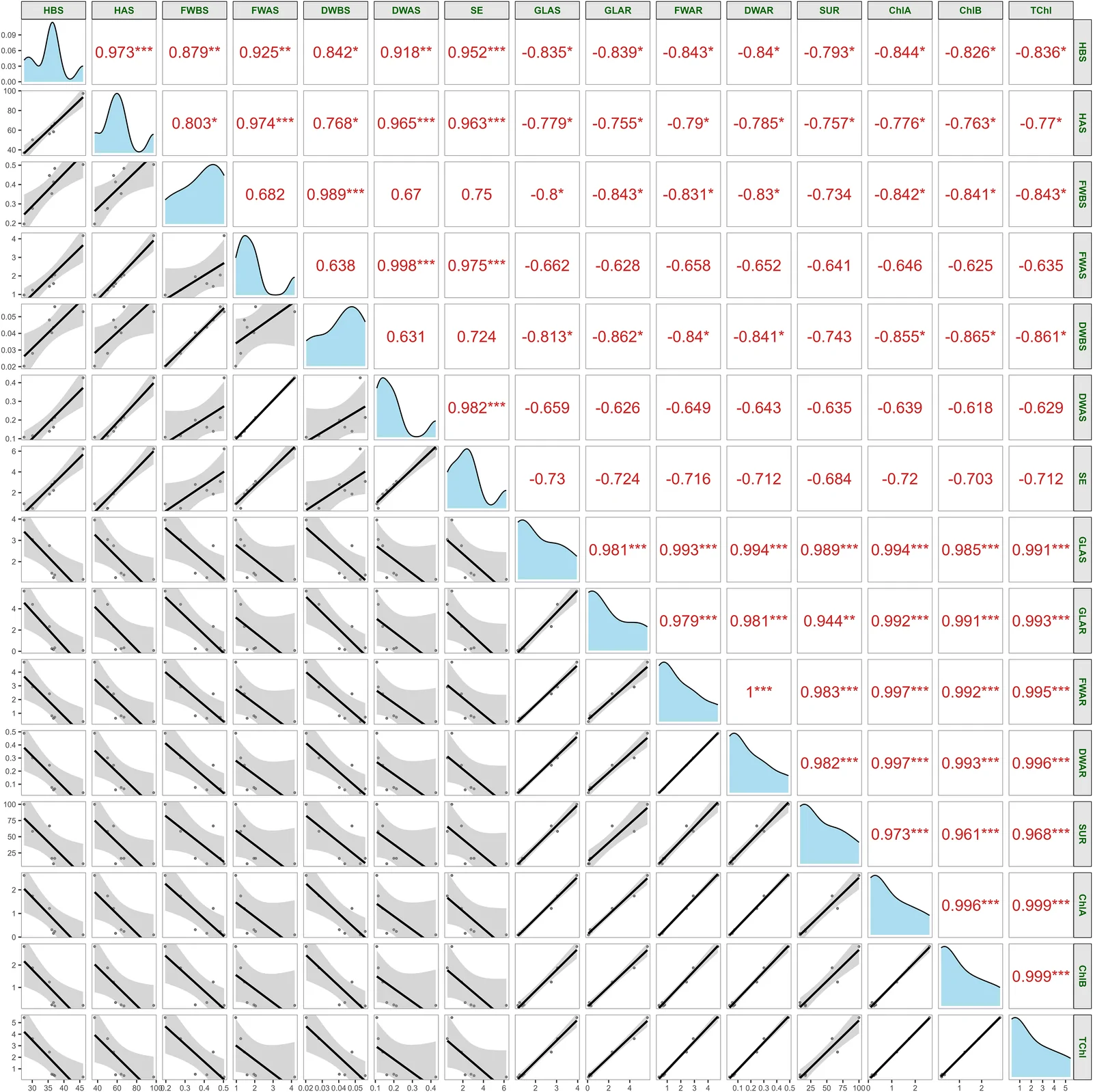
Correlation structure among morphological and physiological traits in rice landraces. HBS: Height Before Submergence, HAS: Height After Submergence, FWBS: Fresh Weight Before Submergence, FWAS: Fresh Weight After Submergence, DWBS: Dry Weight Before Submergence, DWAS: Dry Weight After Submergence, SE: Shoot Elongation, GLAS: Green Leaves After Submergence, GLAR: Green Leaves After Recovery, FWAR: Fresh Weight After Recovery, DWAR: Dry Weight After Recovery, SUR: Survival Percentage, ChlA: Chlorophyll-a, ChlB: Chlorophyll-b, TChl: Total Chlorophyll.

Significant correlations between morphological and physiological characteristics linked to submergence tolerance and recovery were found using the scatterplot matrix and Pearson’s correlation coefficients (Fig 3). Strong positive correlations were found between growth-related traits measured before and after submersion. There was a strong correlation between height before submergence (HBS) and height after submergence (HAS) (r = 0.973***), fresh weight after submergence (FWAS) (r = 0.925***), dry weight after submergence (DWAS) (r = 0.918**), and shoot elongation (SE) (r = 0.952***). HAS also demonstrated strong correlations with SE (r = 0.963***), DWAS (r = 0.965***), and FWAS (r = 0.974***). Vigorous pre-stress growth contributed to improved elongation and biomass retention under flooding, as evidenced by the strong correlation between biomass traits (r = 0.989***) between FWBS with DWBS and (r = 0.998***) between FWAS with DWAS. On the other hand, there were notable negative correlations between recovery-related parameters and growth and elongation traits. Green leaves after submergence (GLAS) (r = –0.835*), green leaves after recovery (GLAR) (r = –0.845*), fresh and dry weight after recovery (FWAR, DWAR; r ≈ –0.84*), survival percentage (SUR) (r = –0.831*), and chlorophyll content (ChlA, ChlB, TChl; r ≈ –0.83* to –0.84*). HAS, FWAS, DWAS, and SE showed similar patterns, indicating that excessive elongation during submergence jeopardizes photosynthetic stability and post-submergence recovery.

There were remarkably strong positive interrelationships between recovery-related traits. GLAS showed a strong correlation with all chlorophyll parameters (r = 0.985*** to 0.994***), GLAR (r = 0.982***), FWAR (r = 0.993***), DWAR (r = 0.994***), and SUR (r = 0.987***). Following recovery, fresh and dry weight showed a nearly perfect correlation (r = 1.000***) and a strong correlation with both chlorophyll traits and survival. High correlations between survival percentage and ChlA (r = 0.970***), ChlB (r = 0.955***), and TChl (r = 0.963***), highlighting the crucial role that photosynthetic pigment retention plays in post-stress survival. The components of chlorophyll had nearly perfect correlation (r ≥ 0.996***). Correlation analysis revealed two functionally distinct trait clusters: recovery, survival, and chlorophyll retention following stress, and elongation and biomass accumulation during submergence. The strong negative correlation between these groups suggests a physiological trade-off whereby excessive shoot elongation under flooding reduces post-submergence recovery capacity and metabolic stability. The significance levels are *P < 0.05, **P < 0.01, and ***P < 0.001.

## 4. Discussion

This study demonstrated that a significant difference exists between the rice landraces in response to submergence, especially in terms of growth, biomass accumulation, survival, and post-submergence recovery. These findings help to evaluate the submergence tolerance of different landraces and also identify the tolerant genotypes among the local landraces in comparison to a known tolerant check. These significant variations among the landraces can be mathematically interpreted via the framework of quiescence versus escape strategies, which are widely considered as the two primary adaptive responses of rice to submergence stress. During submergence stress conditions, rice plants either try rapid shoot elongation to reach the water surface (escape response) or stay dormant and conserve their energy until stress ends (quiescence response) [23]. The results from this study showed that these strategies were strongly followed by the evaluated landraces. Few landraces showed strong growth and rapid shoot elongation under submergence, while others remained dormant and maintained controlled growth and resulted in better recovery, which showed survival under submergence conditions is closely related to energy conservation rather than growth promotion.

### Shoot elongation and survival trade-off: Central mechanism

This study shows a notable inverse relationship between shoot elongation during the submergence and survival after the recovery, which strongly supports the concept of the quiescence model of submergence tolerance. Genotypes such as Chamse 3-mahine achieved the highest shoot elongation during the submergence but ultimately resulted in the highest mortality after the recovery phase, while Sawa Mansuli Sub-1 and Chamse 6-mahine achieved the lowest shoot elongation during submergence but showed significantly higher recovery capacity and resulted in higher plant survival percentages. The experiment by Srividya and Singh [24] also demonstrated that the genotypes that have minimal shoot elongation during submergence achieved a high survival rate and quicker recovery capacity post-submergence. The excessive growth of the genotypes, especially under the hypoxic conditions, can be linked to the action of plant growth hormones, especially gibberellins [25]. The rapid shoot elongation of plants is primarily driven by unregulated gibberellin-mediated growth, which allows plants to escape the submergence by reaching the surface of the water [26]. The strategy followed by plants to escape the submergence by rapid elongation will become unsustainable when submergence is deep and for a longer period of time [27]. Faster elongation during submergence leads to reserve carbohydrate depletion, which is essential for the maintenance of cellular metabolism during poor oxygen conditions [28]. As a result, plants that used excessive reserve carbohydrates for rapid shoot elongation and excessive growth before stress ended lead to higher cellular damage and higher mortality. On the other hand, landraces that show reduced shoot elongation during submergence stress are likely to adopt a quiescence-based approach that is marked by energy conservation and growth suppression [10]. This quiescence-based response is associated with the regulatory function of the gene, which increases the stability of DELLA proteins and inhibits gibberellin-mediated shoot elongation in genotypes harboring *SUB1A*, such as Sawa Mansuli Sub-1 [29]. However, since no molecular genotyping was performed in this study, we cannot be sure that this pathway is responsible for the quiescence-like behavior without further studies. According to Alpuerto et al. [30], the *SUB1A* gene improves DELLA protein stability. As growth repressors, DELLA proteins limit shoot elongation in rice plants when their concentration is high and gibberellin availability is low [31]. For the genotypes that contain *the SUB1A* gene, like Sawa Mansuli Sub-1, this mechanism helps to conserve carbohydrates and reduce gibberellin response during submergence [32]. Moreover, molecular studies are needed to confirm if the same process occurs in local landraces. Chamse 6-mahine showed reduced stem elongation, better green leaf retention, and biomass recovery, which suggests a quiescence-like response to submergence. However, it cannot be confirmed that this response is a SUB1A-DELLA regulated pathway without genetic testing. Sawa Mansuli Sub-1, on the other hand, demonstrated responses consistent with this molecular mechanism and is known to have the *SUB1* gene [33–35]. These results are similar to the [36] experiment, which showed that a major factor influencing rice’s ability to withstand submergence is reduced elongation.

On the other hand, the superior performance of Sawa Mansuli Sub-1, marked by minimal elongation, higher green leaves per plant, and complete survival, coincides well with the quiescence strategy regulated by the *SUB1A* gene [37]. This interpretation is specific to the Sub1 locus-carrying Sawa Mansuli Sub-1. Further genetic confirmation is necessary to determine the molecular basis of similar responses in local landraces. More green leaves indicate high chlorophyll and delayed senescence, which shows that tolerant genotypes are better able to withstand stress conditions [38]. Leaf retention is very important for post-submergence recovery, as it ensures rapid photosynthetic activity to regain its energy. High chlorosis in susceptible genotypes indicates higher oxidative stress and cellular damage prior to long-term submergence [39]. According to Arya et al. [40], genotypes carrying the *SUB1A* gene result in low elongation, higher survival, and better post-submergence recovery. This pattern is clearly reflected in the performance of Sawa Mansuli Sub-1 in this study.

### Biomass dynamics and energy utilization efficiency

The analysis of biomass accumulation across different stages of the experiment provides deeper insight into the energy use strategies of the evaluated landraces. Landraces like Chamse 3-mahine, Rato Bikashi, and Local Basmati showed higher fresh and dry biomass accumulation before submergence, showing strong growth potential under normal conditions. However, this early benefit did not result in increased survival under submergence. During the submergence conditions Chamse 3-mahine continued to show high biomass accumulation, possibly as a result of its fast elongation and active growth metabolism, but this growth occurred as a result of rapid depletion of stored carbohydrates; as a result, this landrace almost failed to survive the recovery phase despite having higher biomass during the stress condition [41,42]. The data clearly shows that high biomass accumulation under stress conditions may indicate inefficient energy utilization rather than tolerance [43]. On the other hand, Sawa Mansuli Sub-1 showed an immediate rise in biomass during the recovery phase, despite having lower biomass accumulation during submergence. Tolerant genotypes prioritize energy conservation under stress conditions and utilize stored resources more effectively after restoring favorable conditions. Such delayed biomass accumulation suggests a strategic allocation of energy toward survival rather than rapid growth during stress [44]. Similarly, Seto Bikashi and Chamse 6-mahine demonstrated moderate biomass accumulation during submergence and better performance during recovery. This suggests a comprehensive strategy that combines partial growth suppression with moderate energy conservation. Overall, these findings demonstrate that submergence tolerance is not determined by total biomass production but rather by the capacity to maintain metabolic stability under stress and the efficiency of energy utilization.

### Leaf retention, chlorophyll stability, and recovery physiology

Leaf retention and chlorophyll content after the submergence stress are the important indicators of submergence tolerance. The result showed that Sawa Mansuli Sub-1, which is the control of this research, had the greatest number of green leaves both during and after submergence, followed by Chamse 6-mahine and Seto Bikashi. On the other hand, susceptible genotypes showed notable chlorosis and leaf senescence, which decreased their photosynthetic capacity. These findings were supported by chlorophyll analysis, which showed that tolerant landraces retained significantly higher levels of chlorophyll content, chlorophyll-a, and chlorophyll-b after the recovery phase [45]. Higher chlorophyll content observed in tolerant genotypes after the seven-day recovery phase is consistent with reduced photosynthetic pigment degradation. However, it reflects recovery after stress rather than pigment retention during submergence itself [46]. Hypoxia in a submerged environment causes reactive oxygen species (ROS) to build up, which can harm cellular structures and accelerate the breakdown of chlorophyll. Therefore, genotypes that recovered with higher chlorophyll content and green leaf viability showed greater capacity to restore their photosynthetic apparatus after stress was relieved [47]. An important part of this process is the regulation of ethylene signaling. Ethylene is the stress hormone that is produced under submergence stress conditions and causes leaf senescence and chlorophyll degradation in plants [48]. Sawa Mansuli Sub-1, which carries the *SUB1* gene, retains the higher number of green leaves and has high chlorophyll content after seven days of recovery periods; this result is consistent with the known *SUB1A*-mediated ethylene suppression responsiveness and delay of senescence [37]. Since chlorophyll was measured after the 7-day recovery period, not immediately after completion of submergence, this value shows post-recovery photosynthetic capacity. Although it is impossible to conclude from the current dataset whether this represents active retention during flooding, accelerated resynthesis during recovery, or both, the noticeably higher chlorophyll content seen in tolerant genotypes is consistent with decreased photosynthetic pigment degradation during submergence. [49]. This results in higher green leaf retention and prolonged photosynthetic activity, which both are necessary for post-submergence recovery [50]. The strong correlation between survival, chlorophyll content, and green leaf retention, which is shown in the result, shows how important it is to preserve physiological functioning under stress. Genotypes that preserve their photosynthetic mechanism are better able to begin growth and restore biomass once normal conditions are restored [51].

### The performance of intermediate and susceptible landraces

Chamse 6-mahine and Seto Bikashi showed moderate levels of shoot elongation, survival, and post-submergence recovery, indicating intermediate genotypic responses. These results suggest that some traditional landraces naturally possess a certain level of submergence tolerance. These landraces retained intermediate chlorophyll content, partial leaf retention, moderate shoot elongation, and comparatively higher survival rates when compared to susceptible genotypes [18]. The idea that submergence tolerance is a quantitatively inherited trait regulated by several genetic and physiological factors rather than being exclusively controlled by the major *SUB1* locus is supported by the observation of such intermediate responses in other conventional germplasm [52]. These genotypes are significant from a breeding standpoint because they could be useful donors of adaptive traits that can be introgressed into cultivars with high yields. Given their moderate performance under stress, targeted selection and genetic enhancement may be able to raise tolerance levels even further. This emphasizes how crucial it is to assess traditional landraces because they frequently contain distinct genetic variability that is lacking in contemporary cultivars [53].

Local Basmati and Rato Bikashi, on the other hand, showed poor performance under submergence stress, even though they grew well under non-stress conditions. These genotypes showed low post-submergence survival rates, excessive shoot elongation, decreased leaf retention, and decreased chlorophyll content. Their incapacity to withstand flooding suggests that they do not have adequate adaptive mechanisms to deal with hypoxic stress. The noticeable elongation of the shoots indicates a dependence on an escape strategy, which is maladaptive when submerged for extended periods of time or in deep water [54]. Additionally, the notably lower chlorophyll content and number of green leaves after recovery in these genotypes suggest weakened physiological stability and a diminished capacity to restore photosynthetic activity after stress [24]. Since high productivity does not necessarily equate to stress resilience, these results show the limitations of assessing genotypes only under ideal circumstances. Certain physiological and genetic adaptations are necessary for survival in harsh environments. Thus, in flood-prone agroecosystems, the need for intentional selection of stress-tolerant genotypes is highlighted by the poor performance of these landraces under submergence stress.

### Principal component and correlation analysis of submergence-related traits

The PCA and correlation analyses provide a useful integrative framework for comprehending the submergence tolerance trait structure in the examined material. According to our findings, the first two principal components accounted for 95.80% of the total variation, suggesting that a comparatively small number of coordinated trait dimensions represented the majority of genotype differences. Chamse 3-mahine occupied a unique position linked to a different response pattern, whereas Sawa Mansuli Sub-1 and Chamse 6-mahine clustered away from the more sensitive genotypes. Submergence tolerance in this study was expressed as a multivariate syndrome rather than an isolated character, as this separation confirms and is consistent with the univariate results.

The reported positive correlations between chlorophyll traits, fresh and dry biomass after recovery, green leaf number after recovery, and survival are highly relevant for screening and biologically consistent. According to studies by S. Singh et al. [55], the maintenance of biomass, non-structural reserves, and chlorophyll is positively correlated with survival under submersion. Basically, this means that in addition to survival itself, chlorophyll retention and post-recovery biomass can be useful secondary selection criteria [56]. The current data shows a negative correlation between excessive shoot elongation and survival, which is equally significant. Sawa Mansuli Sub-1 and Chamse 6-mahine, which elongated the least, had the best survival results in this experiment, while Chamse 3-mahine, the genotype with the greatest elongation, had the lowest survival. This pattern, which is characteristic of quiescence-based tolerance, highlights how restrained elongation is frequently adaptive under full and temporary submergence because it lowers respiratory demand and preserves resources for recovery [57].

Overall, the study’s findings clearly show a strong correlation between submergence tolerance and rice genotypes’ ability to maintain a quiescence-based adaptive strategy, which is characterized by limited shoot elongation, efficient energy conservation, increased chlorophyll retention, sustained green leaf viability, and improved post-submergence recovery. The superior performance of Sawa Mansuli Sub-1 demonstrated the effectiveness of the *SUB1*-mediated tolerance mechanism under total inundation, whilethe comparatively higher survival and recovery rates of Seto Bikashi and Chamse 6-mahine suggest the presence of phenotypic adaptation characteristics in local landraces that are functionally consistent with quiescence-based submergence tolerance. More molecular research is needed to determine whether these characteristics have a genetic foundation similar to or different from the *SUB1*-mediated mechanism. However, susceptible genotypes such as Chamse 3-mahine, Local Basmati, and Rato Bikashi showed excessive shoot elongation, poor post-recovery survival, less green leaf retention after recovery, and the presence of low chlorophyll content following stress, indicating that uncontrolled escape responses are harmful during prolonged deep submersion. The PCA and correlation analyses verified that survival had a positive correlation with biomass recovery, green leaf retention, and chlorophyll stability, but shoot elongation had a negative correlation with tolerance. Therefore, the current study highlights that rapid vegetative growth under stress is not as significant in determining submergence tolerance as recovery efficiency and physiological stability [58]. Furthermore, the finding of moderately tolerant local landraces provides significant potential for future breeding initiatives aimed at developing climate-resilient rice cultivars for Nepal’s flood-prone regions and other agro-ecological conditions. As measurement of biomass and biochemical required destructive sampling, only three replications remained for recovery-phase analysis. This may have contributed to the comparatively high coefficients of variation seen for several recovery features and decreased the statistical power for these variables. However, all genotypes were subjected to the same sampling procedure and under the same experimental conditions; statistically significant genotypic differences were found. The stability and generalizability of these results would be further strengthened by future research using bigger sample sizes.

## 5. Conclusion

This study explores phenotypic variation in local rice landraces’ responses to complete submergence, highlighting that submergence tolerance involves coordinated differences in growth dynamics, biomass, chlorophyll retention, and recovery post-stress. However, these local landraces’ survival rates (58.3% for Chamse 6-mahine and 66.7% for Seto Bikashi) remain moderate and fall short of the 100% survival seen in Sawa Mansuli Sub-1, suggesting that more genetic improvement is required before these landraces can be used in production environments that are vulnerable to flooding. Conversely, escape-type genotypes like Chamse 3‑mahine manifested unchecked shoot elongation, leading to chlorosis, rapid carbohydrate depletion, and eventual highest mortality, illustrating the drawbacks of uncontrolled growth in deep inundations. Multivariate analyses indicated that submergence tolerance encompasses a complex trait syndrome, correlating positively with post-recovery biomass, green leaf retention, and chlorophyll stability, while negatively with shoot elongation. The findings suggest using chlorophyll maintenance and recovery biomass as secondary selection criteria in submergence tolerance screening programs, alongside survival. Notably, Chamse 6-mahine and Seto Bikashi, along with the tolerant check Sawa Mansuli Sub-1, showed phenotypic profiles consistent with quiescence-based submergence tolerance and represent potentially useful genetic resources for future breeding programs, subject to molecular characterization and field validation. Ultimately, the research underscores the importance of breeding for traits that promote physiological stability and recovery efficiency under submergence in flood-prone agroecosystems, rather than merely focusing on pre-stress vigor or rapid elongation. Future studies should combine precise phenotyping with molecular analyses of critical loci, like *SUB1*, to unravel the genetic architecture of tolerance and advance climate-resilient rice cultivars suitable for environments like Nepal.
